# Growth performance and small intestinal morphology of native chickens after feed supplementation with tryptophan and threonine during the starter phase

**DOI:** 10.14202/vetworld.2020.2765-2771

**Published:** 2020-12-24

**Authors:** Charles V. Lisnahan, Oktovianus R. Nahak

**Affiliations:** Department of Animal Husbandry, Faculty of Agriculture, University of Timor, East Nusa Tenggara 85613, Indonesia

**Keywords:** growth performance, native chickens, small intestinal morphology, starter phase, threonine, tryptophan

## Abstract

**Background and Aim:**

The amino acid content of feed can affect growth performance of poultry during the first 6 weeks of life or the starter phase. Unlike for broiler and layer chickens, there is no information concerning standard requirements for tryptophan and threonine during the starter phase. This study aimed to determine the amount of threonine and tryptophan that should be supplemented in chicken feed to maximize growth performance and small intestinal morphology of native chickens during the starter phase.

**Materials and Methods:**

A total of 128 day-old native chickens were divided into four treatment groups with four replications based on a completely randomized design. The treatment diets were as follows: T_0_ (control feed); T_1_ (T_0_+0.10% L-tryptophan+0.35% L-threonine); T_2_ (T_0_+0.17% L-tryptophan+0.68% L-threonine); and T_3_ (T_0_+0.25% L-tryptophan+1.00% L-threonine).

**Results:**

The feed intake was highest for the T_2_ and T_3_ groups (123.06 and 124.18 g/bird/week, respectively). The T_3_ group had the highest body weight gain (49.35 g/bird/week) and carcass weight (201.44 g/bird) relative to the other groups, while the T_2_ and T_3_ groups showed similar, significant (p<0.05) increases in feed conversion ratio (2.57 and 2.51, respectively) and carcass percentage (60.88 and 60.99%/bird, respectively) compared to the other groups. This study showed villi height, crypt depth, and villi width of duodenum, the highest jejunum and ileum of T3 (1109.00±27.26, 1325.50±75.00, 1229.50±101.68, 225.50±17.52, 236.00±24.81, 219.75±17.25, 192.25±14.41, 191.75±4.79, and 184.75±6.40, respectively) compare to other treatment.

**Conclusion:**

These results indicate that supplementation of feed with 0.17% L-tryptophan and 0.68% L-threonine positively affected the growth performance and small intestinal morphology of native chickens during the starter phase.

## Introduction

Protein in the starter phase (0-6 weeks of age) is critical for the survival of native chickens. Amino acids as a constituent of proteins play roles in the growth and physiological functions of chickens. Micronutrients, especially balanced amino acids, can promote maximum growth in native chickens. Protein derived from fish and soybean meal included in diets for native chickens can be very expensive. For economical supplementation to address deficiencies in specific amino acids in poultry feed, synthetic amino acids have been added to chicken feed, particularly beginning in the early 1990s.

In a previous study, Lisnahan *et al*. [1] showed that growth performance and feed efficiency of starter phase native chickens could be improved by the addition of the critical amino acids methionine and lysine. Threonine and tryptophan are two other limiting amino acids in chicken diets. Threonine is involved in bone formation and a variety of physical systems, including the immune, nervous and digestive systems, and the liver. This amino acid also is important for maintaining protein levels and fat metabolism [2]. Tryptophan is a serotonin trigger that stimulates niacin production, appetite, and feed efficiency and also promotes increases in body weight [3,4].

Unlike for broiler and layer chickens, there is no information concerning standard requirements for tryptophan and threonine during the starter phase. This study aimed to determine the amount of threonine and tryptophan that should be supplemented in chicken feed to maximize growth performance and small intestinal morphology of native chickens during the starter phase.

## Materials and Methods

### Ethical approval

The study protocol was approved by Animal Ethics Committee of the Animal Husbandry Program Study, Agriculture Faculty, Timor University, Indonesia.

### Study period, location, Animal, and feed preparation

This study was conducted in Kefamenanu in the East Nusa Tenggara province of Indonesia between February 2020 and April 2020. A total of 128 day-old chicks hatched in Kefamenanu, East Nusa Tenggara and weighing on average 34 g were used in the study. Chicks were vaccinated at 3 and 21 days old with ND1 and ND2 vaccines, respectively. Feed was obtained from farmers, with the exception of fish meal, soybean meal, amino acids, and vitamin and mineral premixes, which were purchased from a poultry shop. The cage units used were 100 cm×100 cm×70 cm and 16 individual cages were contained within a larger cage that was 6 m×9 m×5 m. Each cage unit had eight chickens.

### Dietary treatment and feeding duration

Chicks were randomly assigned to one of four groups in a completely randomized design (CRD) experiment involving four treatments: T_0_ (control feed), T_1_ (T_0_+0.10% L-tryptophan+0.35% L-threonine, T_2_ (T_0_+0.17% L-tryptophan+0.68% L-threonine), and T_3_ (T_0_+0.25% L-tryptophan+1.00% L-threonine). The feed ingredients and nutrient composition of each treatment are shown in Table-1. Feed and water were available *ad libitum* during the study period. Feed was given every morning and evening. Feed was weighed daily and chickens were weighed weekly. After 6 weeks, the chickens were sacrificed and the carcass weight and small intestinal morphology were determined for 32 birds.

**Table-1:** Composition (%) and nutrient content (% dry matter) of experimental diets during the starter phase (1-6 weeks).

Ingredients	Treatments (%)			

	T_0_	T_1_	T_2_	T_3_
Yellow corn	55.00	55.00	55.00	55.00
Rice bran	28.20	27.75	27.35	26.95
Soybean meal	8.00	8.00	8.00	8.00
Fish meal	7.00	7.00	7.00	7.00
Mineral premix	0.40	0.40	0.40	0.40
Vitamin premix	0.34	0.34	0.34	0.34
Dl-methionine	0.27	0.27	0.27	0.27
L-lysine HCl	0.79	0.79	0.79	0.79
L-tryptophan	0	0.10	0.17	0.25
L-threonine	0	0.35	0.68	1.00
Total	100.00	100.00	100.00	100.00
Calculated nutrient content (%)				
Metabolized energy (kcal/kg)	2962.85	2952.82	2943.92	1935.87
Crude protein	16.75	16.72	16.68	16.65
Ether extract	6.01	5.97	5.94	5.90
Ash	8.08	8.04	8.00	7.96
Crude fiber	6.97	6.91	6.85	6.79
Methionine	0.30	0.30	0.30	0.30
Lysine	0.85	0.85	0.85	0.85
Tryptophan	0.03	0.13	0.20	0.28
Threonine	0.05	0.40	0.72	1.05
Calcium	1.62	1.62	1.62	1.62
Phosphorus available	0.58	0.58	0.58	0.58

### Small intestinal morphology measurement

The small intestinal morphology measurement comprise was villi height, villi width, and crypt depth. Villi height, villi width, and crypt depth are measured in stages:

#### Chicken intestinal sample preparation

The small intestinal segments prepared as samples are the duodenum, jejunum, and ileum. Each part was taken 2 cm pieces and fixed in 10% buffer formalin, soaked for 24-48 h, and then made preparations.

#### Preparation making

The way of making hematoxylin-eosin preparations, each piece of tissue is hydrated through a series of alcohol whose concentration is increasing (70, 80, 90, and 95%). The samples were transferred one by one to each alcohol concentration and allowed to soak for about 10 s. Then, the sample is inserted into xylol and finally immersed in paraffin. The sample was sliced thin using a microtome for hematoxylin-eosin staining. The histological preparations that were ready in the glazed object were observed and measured using a computer microscope.

#### Shooting

The object of the sample was viewed and determined using an Olympus BX 51 microscope equipped with an Olympus DP 12 projector adjusted to 10 times magnification. Morphological images appeared on the JVC TMH 1750 C monitor. After finding the intestinal morphology as expected, all the preparations to be measured were taken. Minimum measurements of 3 times per slide are made for parameters.

#### Measurement steps for villi height, villi width, and depth of Lieberkuhn crypt

The measurements of villi height, villi width, and depth of Lieberkuhn crypt were done using a flat screen computer with the Microsoft Office Picture Manager program at 40% magnification. At first, the standard size of μm is determined with the help of a computer, namely, how much the magnification value used or desired is converted into units of length (μm). The μm unit number obtained was then used as a standard in measuring the villi height, villi width, and the depth of the crypt displayed on the monitor screen.

### Data collection and analysis

Several parameters of native chickens were measured including feed intake, body weight gain, feed conversion, carcass weight, and carcass percentage. Bodyweight gain was the difference in weight between the final body weight and the initial body weight. The feed conversion ratio is calculated based on weight gain divided by feed intake. Carcass weight was obtained after being reduced by blood, feathers, head and neck, shank, internal organs, and abdominal fat. The small intestinal morphology observed was villi height, crypt depth, and villi width of duodenum, jejunum, and ileum. The data were subjected to an analysis of variance based on the CRD and Duncan’s test (IBM SPSS Statistics 22).

## Results

### Body weight gain

**Figure-1:**
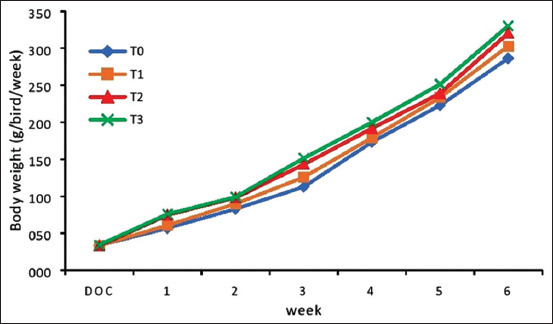
Relationship between diet treatment and body weight of native chickens during the starter phase (1-6 weeks).

Statistical analysis showed significant increases in body weight for all experimental groups during the starter phase (p<0.05; Table-2). The average weight gain was highest for the T_3_ group (0.25% L-tryptophan and 1.00% L-threonine) followed by the T_2_ (0.17% L-tryptophan and 0.68% L-threonine) and T_1_ group (0.10% L-tryptophan and 0.35% L-threonine), whereas birds in the T_0_ group (no amino acid supplementation) gained the least weight (Figure-1). The T_2_ and T_3_ groups had similar percentages of body weight gain, 6.60 and 6.69%, respectively. The highest rate of weight gain for native chickens was seen for the T_3_ group at 49.35±0.97 g/bird/week, but this increase did not significantly from that seen for the T_2_ group (47.87±1.15 g/bird/week).

**Table-2:** Growth performance of native chickens during the starter phase (1-6 weeks).

Parameters	Treatments			

	T_0_	T_1_	T_2_	T_3_
Feed intake (g/bird/week)	109.75±4.43^c^	116.57±4.93^b^	123.06±4.01^a^	124.18±3.09^a^
Body weight gain (g/bird/ week)	42.09±1.67^c^	44.87±2.18^b^	47.87±1.15^a^	49.35±0.97^a^
Feed conversion ratio	2.61±0.11	2.60±0.02	2.57±0.02	2.52±0.06
Carcass weight (g/bird)	167.02±5.47^c^	181.88±6.01^b^	195.04±5.20^a^	201.44±3.55^a^
Percent carcass (%/bird)	58.31±0.83^b^	60.05±0.33^a^	60.88±0.80^a^	60.99±0.66^a^

^a,b,c,d^Different superscript on the same line indicates significant difference (p<0.05)

### Feed intake

**Figure-2:**
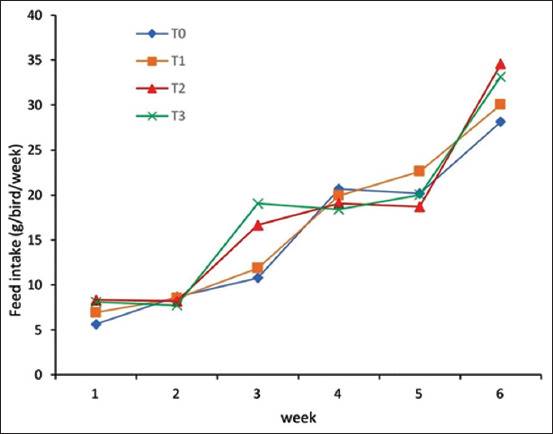
Relationship between diet treatment and feed intake of native chickens during the starter phase (1-6 weeks).

Native chickens had significant feed intake during the starter phase (p<0.05; Table-2). The average feed consumption was highest for the T_3_ group followed by the T_2_ and T_1_ groups. The T_0_ group with no supplementation consumed the least feed (Figure-2). Supplementation with the lowest levels of L-tryptophan and L-threonine in the T_1_ group was associated with an increase of 6.21% relative to the T_0_ group, and the intake increased by another 5.57% for the T_2_. A slight, but not significant, increase over that for T_2_ was seen for T_3_.

### Feed conversion ratio

**Figure-3:**
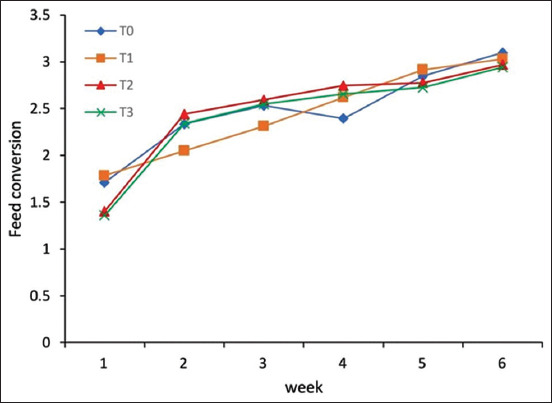
Relationship between diet treatment and feed conversion ratio of native chickens during the starter phase (1-6 weeks).

Differences in the feed conversion ratio of native chickens at the beginning of the starter phase were not significant (Figure-3). The average conversion of native chicken feed was highest for birds in the T_0_ treatment group, followed by those in T_1_, T_2_, and T_3_ groups, respectively (Table-2 and Figure-3). This result suggests that tryptophan and threonine supplementation does not affect the feed conversion ratio during the starter phase.

### Carcass weight

The carcass weight of native chickens showed significant differences among the treatment groups (p<0.05; Table-2). Birds in the T_3_ group had the highest carcass weight and percentage, followed by the T_2_, T_1_, and T_0_ groups. The T_1_ group showed an 8.89% increase in carcass weight over that for the T_0_ group. Further increases in the amount of tryptophan and threonine supplementation increased the carcass weight by another 3.35% for the T_2_ group, which was similar to that for the T_3_ group.

### Small intestinal morphology

The villi height, depth of crypt, and villi width of duodenal, jejunum, and ileum are presented in Table-3. The data showed that the villi height, crypt depth, and the villi width of the duodenal, jejunum, and ileum at the end of the starter phase of native chickens (6 weeks) were significantly different (P<0.05). Supplementation of 0.10% l-tryptophan and 0.35% l-threonine (T_1_) increased the duodenal and ileum villi height by 11.93% and 21.15% compared to T_0_. At the 0.17% l-tryptophan and 0.68% l-threonine (T_2_) levels, there was an increase in villi height in the duodenum, jejunum, and ileum by 5.97%, 17.80%, and 22.79% compared to T1. When increased at the 0.25% l-tryptophan and 1.00% l-threonine levels, duodenal villi height increased by 7.77% compared to T_2_.

**Table-3:** Average of small intestine morphology size of native chickens at 6 weeks age.

Characteristic of intestine	Small intestine part	Treatments			

		T_0_	T_1_	T_2_	T_3_
Villi height	Duodenum	867.50±44.36^d^	971.00±14.45^c^	1029.50±26.94^a,b^	1109.00±27.26^a^
	Jejunum	989.50±42.05^b^	1069.00±42.15^b^	1259.25±154.14^a^	1325.50±75.00^a^
	Ileum	766.00±11.62^c^	928.00±41.59^b^	1139.50±130.16^a^	1229.50±101.68^a^
Crypt depth	Duodenum	166.00±16.02^b^	186.00±13.51^b^	210.75±12.66^a^	225.50±17.52^a^
	Jejunum	177.00±4.69^c^	199.75±6.95^b,c^	220.25±16.98^a,b^	236.00±24.81^a^
	Ileum	143.75±4.92^c^	177.75±6.29^b^	207.50±13.28^a,b^	219.75±17.25^a^
Villi width	Duodenum	136.00±9.83^c^	153.00±11.04^b^	169.00±4.24^a,b^	192.25±14.41^a^
	Jejunum	134.00±5,60^c^	154.00±15.38^b^	178.25±6.13^a^	191.75±4.79^a^
	Ileum	122.50±5.07^c^	146.75±15.11^b^	172.50±3.70^a^	184.75±6.40^a^

^a,b,c,d^Different superscript on the same line indicates significant difference (p<0.05)

Supplementation of 0.10% l-tryptophan and 0.35% l-threonine (T_1_) increased the ileum crypt depth by 23.65% compared to T_0_. When increased at the level of 0.17% l-tryptophan and 0.68% l-threonine (T_2_), there was an increase in the crypt depth of the duodenal by 13.31% compared to T_1_. At the level of 0.25% l-tryptophan and 1.00% l-threonine, the crypt depth of jejunum increased by 18.15% compared to T1, while for ileum, there was an increase of 5.90% compared to T_2_.

Supplementation of 0.10% l-tryptophan and 0.35% l-threonine (T_1_) increased the villi width of the duodenal, jejunum, and ileum by 12.50%, 14.92%, and 19.80% compared to T_0_. Changes in the villi width of the jejunum and ileum also occurred at the level of 0.17% l-tryptophan and 0.68% l-threonine (T_2_) of 15.75% and 17.55% compared to T1. At the 0.25% l-tryptophan and 1.00% l-threonine level, the duodenal villi width increased by 32.56% compared to T2, while the jejunum and ileum were not significant.

## Discussion

Feed supplementation with L-tryptophan and L-threonine increased the body weight of native chickens. In a previous study, Lisnahan *et al*. [1] reported that 6-week-old native chickens had an average weight of 284.61 g/bird when given feed supplemented with Dl-methionine and L-lysine, but lacking L-tryptophan and L-threonine. In the present study, birds given a diet supplemented with 0.25% L-tryptophan and 0.85% L-threonine had an average body weight of 330.29 g/bird. For chickens, after methionine and lysine, threonine and tryptophan are the next most critical amino acids [3,5].

Opoola *et al*. [5] showed that the addition of 0.23-0.31% tryptophan to feed increased the body weight of broiler chickens, whereas Wei *et al*. [6] reported that broiler chicken weight gain could be achieved by the addition of 0.23% tryptophan in feed under feed restriction. Threonine and tryptophan supplementation not only increases body weight, but also accelerates growth to maximize growth of chickens. Cafe and Waldroup [7] found that chicken body weight could be influenced by the availability and balance of amino acids in the feed. In addition to methionine and lysine, threonine and tryptophan play roles in metabolic and physiological functions [3,8]. Wen *et al*. [9] suggested that threonine, lysine, methionine, valine, and isoleucine are important muscle components and deficiencies in these amino acids are always associated with reduced weight. High levels of tryptophan positively affected systemic immune responses and growth performance in poultry [10,11]. Meanwhile, niacin-deficient poultry feed affects the action of tryptophan by ameliorating weight gain and modulating feed consumption [12].

Feed intake and body weight are influenced by threonine [13,14], while tryptophan regulates protein biosynthesis and, in particular, is involved in increasing muscle mass and stimulating immune responses. Tryptophan can promote stability of intracellular proteins that contribute to enhanced growth and antibody production. In addition, tryptophan is a precursor of serotonin that is involved in mental function [3,15,16]. Studies by Wei *et al*. [6] and Duarte *et al*. [17] showed that tryptophan is indeed important for protein synthesis and as a serotonin precursor that stimulates feed intake. In poultry, tryptophan is converted into 5-hydroxytryptophan molecules that play a role in production of both serotonin and melatonin in the brain to control stress and increase metabolism [18]. Wen *et al*. [9] showed that feed consumption, behavior, growth, immunity, protein synthesis, and intestinal integrity of livestock can be regulated by tryptophan supplementation. Tryptophan is prominent among essential amino acids due to these critical roles in protein synthesis and serotonin production that together stimulate feed consumption and promote growth [17].

L-threonine supplementation enhances immunity, antioxidant capacity, and intestinal health of broilers in the starter phase. In particular, immunoglobulins, including immunoglobulin A (IgA), immunoglobulin G, and secreted IgA involved in immune responses and alleviation of stress are affected by threonine levels [13,19]. Rezaeipor *et al*. [20] reported that threonine improved the morphological characteristics of the small intestinal in broiler chickens and these characteristics are reflected by an increased growth rate. Chickens fed a diet deficient in threonine have reduced nutrient absorption in the digestive tract. Estalkhzir *et al*. [13] and Azzam *et al*. [21] also showed that threonine is important for maintaining mucosal stability in the digestive tract that leads to increased appetite, digestion, and nutrient absorption that translate to increased body weight. Lisnahan and Nahak [4] and Ahmadi and Golian [22] demonstrated that threonine affects bone formation and plays multiple roles in immune system function and fat metabolism, as well as various physiological systems, including the liver, nervous system, and digestive tract.

Native chickens given feed with L-tryptophan and L-threonine supplementation had increased body weight and feed consumption compared to birds without supplementation. Lisnahan *et al*. [1] reported that during the starter phase (0-6 weeks), feed conversion decreased with increasing levels of methionine and lysine in feed. Here, we observed no effect of L-tryptophan and L-threonine through the starter phase. However, the feed conversion ratio tended to be higher for native chickens given feed supplemented with L-tryptophan and L-threonine relative to unsupplemented feed (Figure-3). This result suggests that feed conversion is impacted by threonine and tryptophan. Feed conversion is influenced by genetics, type of feed, and feed additives used, in addition to livestock management methods and environmental factors such as temperature [23]. Threonine and tryptophan supplementation balances dietary amino acids to optimize amino acid ratios and protein content of feed that together can reduce the amount of added protein, which can be expensive, needed to maximize growth gains of poultry.

Carcass weight correlates to body weight. Tamzil [24] reported that the percentage of native chicken carcass at the end of the starter phase (6 weeks) was 61.39%. Adequate amounts and the balance of amino acid in feed consumed by chickens can affect the production performance and feed use efficiency [1,25]. Important metabolic processes such as uric acid formation and protein synthesis involve threonine [21]. Suryawan *et al*. [26] reported that the increase in carcass weight was influenced by the rate of formation of new tissue compared to the rate of tissue damage. Tryptophan and threonine are both important for protein synthesis and increased muscle mass to increase body weight, especially muscles that comprise the carcass [15,16].

Kidd and Hackenhaar [27] found that tryptophan deficiency not only affects carcass quality by affecting protein synthesis but also impairs the synthesis of important neurotransmitters such as serotonin and melatonin. Leeson and Summers [28] reported that amino acid availability in chicken feed affects growth, production, and efficiency of feed use that is related to tissue formation and, in turn carcass weight. Furthermore, Zhai *et al*. [29] stated that the addition of lysine in feed and several amino acids in the form of soluble protein enhanced the water retention capacity and pH of breast meat, while also reducing protein denaturation and increasing levels of methionine and threonine. Thus, lysine and, in particular, threonine are important for the formation of breast meat, which represents a major component of the carcass [30].

The increase of villi size (height, crypt depth, and villi width) was positively correlated with feed consumption and growth (Table-2). Chicken growth depends on digestion and absorption of nutrients related to the morphological and functional development of the small intestine. An increase of villi size is associated with an increase in digestive function for nutrient absorption. Awad *et al*. [31] stated that the increase of villi height in the chicken intestine is parallel to the increase of digestive and absorption functions as well as a smooth expression of the nutrient transport system throughout the body.

Increasing crypt depth causes absorption of more nutrients into the bloodstream, causing the increasing of growth and efficiency of feed used to be better. It can also be seen that at T3, body weight gain and feed consumption are better. Sun *et al*. [32] stated that the development of chicken intestinal villi is related to intestinal function and growth of the chicken. The ability to digest and absorb nutrients is influenced by the surface area of the intestinal epithelium, the number of folds, and the number of villi and microvilli that expand the absorption field [31]. As a result, the development of the digestive tract, including the intestine is also better. The size of the intestine is larger and longer (number, height, and width of the villi) as a place for better absorption of nutrients into all body tissues. Fitasari [33] stated that one of the parameters that can be used to measure the quality of growth is the intestinal morphological structure. The intestinal villi are a place for absorption of nutrients, the wider the villi the more nutrients are absorbed which ultimately has an impact on the growth of the body’s organs. Awad *et al*. [31] stated that an increase in the height and width of the villi in chicken intestines is closely related to an increase in digestive function and absorption function due to the wider absorption area and is an expression of the smooth transportation system for nutrients throughout the body’s tissues. Chickens are given amino acids which can improve digestibility in the ileum and can also increase the digestibility of other amino acids such as isoleucine, phenylalanine, valine, aspartic acid, and tyrosine [34].

Azzam *et al*. [19] stated that threonine increases humoral immune response by increasing globulin levels and antioxidant levels in chickens. In addition, threonine helps maintain the integrity of the intestinal mucosal barrier and thus improves nutrient absorption. Azzam *et al*. [21] and Estalkhzir *et al*. [13] found that adding threonine to the diet of broilers increased productivity in terms of body weight, feed conversion, relative breast weight, and thigh weight. Among the essential amino acids, threonine plays an important role in the maintenance of intestinal barrier integrity and mucin synthesis [20,35,36].

## Conclusion

Feed intake, body weight gain, carcass weight, carcass percentage, villi height, crypt depth, and villi width of small intestine of native chickens during the starter phase were increased by supplementation with feed containing increasing amounts of L-threonine and L-tryptophan. In particular, supplementation with 0.17 L-tryptophan and 0.68% L-threonine produced optimal growth performance of native chickens during the starter phase.

## Authors’ Contributions

CVL compiled the experiment ideas and designed the work, collected the data, supervised the work, analyzed the data, and drafted the manuscript. ORN designed the work, the laboratory work, and supervised the work. CVL and ORN revised and agreed to the final manuscript. All authors read and approved the final manuscript.
